# Cytotoxicity of Ag, Au and Ag-Au bimetallic nanoparticles prepared using golden rod (*Solidago canadensis*) plant extract

**DOI:** 10.1038/s41598-019-40816-y

**Published:** 2019-03-12

**Authors:** Tarryn L. Botha, Elias E. Elemike, Suranie Horn, Damian C. Onwudiwe, John P. Giesy, Victor Wepener

**Affiliations:** 1 Water Research Group, Unit for Environmental Sciences and Management, Potchefstroom Campus, North-West University, Private Bag X6001, Potchefstroom, 2520 South Africa; 2 Material Science Innovation and Modelling (MaSIM) Research Focus Area, Faculty of Agriculture, Science and Technology, North-West University, Mafikeng Campus, Private Bag X2046, Mmabatho, 2735 South Africa; 3 Department of Chemistry, School of Mathematics and Physical Sciences, Faculty of Agriculture, Science and Technology, North-West University, Mafikeng Campus, Private Bag X2046, Mmabatho, 2735 South Africa; 4 Department of Chemistry, College of Science, Federal University of Petroleum Resources, P.M.B, 1221 Effurun, Delta State Nigeria; 5 Department of Veterinary Biomedical Sciences and Toxicology Centre, University of Saskatchewan, Saskatchewan, Canada; 6 Department of Zoology, and Center for Integrative Toxicology, Michigan State University, East Lansing, MI USA; 7 School of Biological Sciences, University of Hong Kong, Hong Kong, SAR China; 8 State Key Laboratory of Pollution Control and Resource Reuse, School of the Environment, Nanjing University, Nanjing, People’s Republic of China

## Abstract

Production and use of metallic nanoparticles have increased dramatically over the past few years and design of nanomaterials has been developed to minimize their toxic potencies. Traditional chemical methods of production are potentially harmful to the environment and greener methods for synthesis are being developed in order to address this. Thus far phytosynthesis have been found to yield nanomaterials of lesser toxicities, compared to materials synthesized by use of chemical methods. In this study nanoparticles were synthesized from an extract of leaves of golden rod (*Solidago canadensis*). Silver (Ag), gold (Au) and Ag-Au bimetallic nanoparticles (BNPs), synthesized by use of this “green” method, were evaluated for cytotoxic potency. Cytotoxicity of nanomaterials to H4IIE-*luc* (rat hepatoma) cells and HuTu-80 (human intestinal) cells were determined by use of the xCELLigence real time cell analyzer. Greatest concentrations (50 µg/mL) of Ag and Ag-Au bimetallic were toxic to both H4IIE-*luc* and HuTu-80 cells but Au nanoparticles were not toxic. BNPs exhibited the greatest toxic potency to these two types of cells and since AuNPs caused no toxicity; the Au functional portion of the bimetallic material could be assisting in uptake of particles across the cell membrane thereby increasing the toxicity.

## Introduction

Recently, nanotechnology has become an intensely researched area of and nanoproducts are widely gaining uses, especially in electronics, health care, cosmetics and medicine. One question is, how safe are nanomaterials? In assessing cytotoxicity of nanomaterials, one aspect is to determine potencies under various conditions of the cell cultures, such as temperature, pH and nutrient concentrations^1^.

Gold (Au) and silver (Ag) nanoparticles are the most studied noble metals and they are increasingly being applied in various biological treatments. Silver has been applied as antimicrobial agents, whereas gold nanoparticles have shown promise in diagnosis and therapy of cancer^2,3^. Au-NPs absorb visible light and within picoseconds, deliver wavelength-specific energies with targeted precision and efficiency. Thus, they can be applied in light-mediated clinical treatments (photodynamic therapies), for which bimetallic alloy NPs could be seen to exhibit better functionalities. The colour of Au nanoparticles, which is in the visible region of the spectroscopy, have ability to bind with biological molecules or ligands which aids in bioimaging and other biomedical applications.

These noble nanometals exist in various structures, such as nanospheres, nanocages, nanorods, nanoflowers, nanopolygons and their functions vary based on produced structures^1,4^. Various morphologies offer interesting possibilities of diffusion, surface interaction with target molecules or organisms thereby directing their roles actively. Since sizes of nanomaterials are in the nanometre range, they are able to penetrate cells, a property that has been utilized in cell targeting.

There are three major methods of synthesizing nanoparticle: physical, chemical and biological, but the most used and conventional method is the chemical approach. Chemical synthesis of NPs results in NPs being less toxic (e.g. Au) or equally toxic (e.g. Ag) relative to bulk chemicals^5,6^. When used in cellular applications, due to gradual releases of chemicals, used during syntheses, chemical-based syntheses have disadvantages due to toxicities to cells. Development and uses of effective alternatives, such as nanoparticles produced by use of extracts of plants might exhibit lesser toxic potencies. Plant materials contain active pharmacological ingredients, which not only serve as reducing agents, but can also act as capping agents for NPs and as a result intensify their biomedical efficacies.

Furthermore, the use of biomolecules as reductants offers significant advantages over other similar protecting agents^7^. Au and Ag have a long history of antimicrobial and anti-infective properties that exceed that of their metal ions and as such synergistic actions of NPs containing these two metals, would inherently surpass previously existing materials of similar action with due to lesser toxicity exhibit excellent biocompatibility. Also, to avoid or minimize toxicity to cells and environments, costs of synthesis and dangers involved in handling chemical reducing agents, more eco-friendly methods for syntheses of metal NPs were preferred. Since extracts of the angiosperm *Solidago canadensis* has been used traditionally for several medicinal applications relating to antimicrobial and antioxidant effects, it was hypothesized that it could be used during phytosynthesis of nanomaterials^8^.

Application of biogenic phytosynthesis to produce NPs has been proposed as a more biocompatible, alternative to chemical syntheses^9^. Phytosynthesised AgNPs^10^ and AuNPs^11^ exhibited lesser toxic potencies than did NPs produced via chemical reactions. However, there were no data on comparative toxicities of monometallic and bimetallic photosynthesized NPs, which hampered assessment of potential hazards of NPs. In this study, cytotoxicity of Au, Ag, and Au-Ag bimetallic alloy NPs produced by use of extracts of leaves of *S. canadensis* were determined and results used to assess potential effects of these NPs on humans and wildlife^12^. H4IIE*-luc* rat hepatoma cells were used as an indication of a detoxification response and the HuTu-80 cells (HTB-40™) isolated from human intestine were used to indicate uptake by this tissue. Rat liver and human intestine equivalents of normal cells were not included in this study due to availability. It was hypothesised that advanced physicochemical properties exhibited by the novel, monometallic and bimetallic NPs might influence applications in drug delivery, medical theranostics and *in vivo* imaging.

## Materials and Methods

### Characterization

#### Syntheses of nanomaterials using plant extract

Leaves from the plant *S. canadensis* (golden rod) of which identification was confirmed by a plant taxonomist; were collected from a botanical garden in Mafikeng, North West Province, South Africa. In preparation for processing, leaves were washed using double distilled water to remove sand and debris and were dried at room temperature (22–26 °C) under air for three weeks before being ground using a pestle and mortar. An aqueous extract was prepared by heating approximately 2 g of ground plant extract in 100 mL of distilled water at 80–85 °C and filtered immediately through Whatmann filter paper. The filtrate was allowed to cool to 25 °C and used for synthesis of NPs. Silver nitrate (AgNO_3_) and Gold (III) chloride hydrate (HAuCl_4_.xH_2_O) (Sigma Aldrich, Darmstadt) were used to synthesize gold and silver nanoparticles; where the plant extract (50 mL) was added to 500 mL of aqueous 1 mM HAuCl_4_.xH_2_O and AgNO_3_ salt respectively. Samples were heated between 70–80 °C for a period of 1 hour. Solutions were sampled at different intervals as the reaction underwent a colour change, samples were analysed for the appearance of plasmon bands monitored by use of an UV-Vis spectrophotometer (UV-1901 Agilent Technology, Cary series UV-vis spectrometer, USA). A similar process was followed for Ag-Au bimetallic nanomaterials, however 250 mL of each ionic salt was added *in situ* to the 50 mL of plant extract. Periodic changes in colour were seen due to formation of plasmon bands, which were also confirmed by use of UV-vis spectroscopy.

#### Transmission electron microscopy

Transmission electron microscopy (TEM) was performed by applying one drop of the prepared nanomaterials (AuNPs, AgNPs and Ag-Au bimetallic NPs) onto a carbon coated copper grid and allowed to settle for three minutes. The grid was allowed to dry and TEM was performed using of a model JEOL2100 instrument fitted with a LaB 6 electron gun. Images were captured using a Gatan Ultrascan digital camera.

#### Characterization in exposure medium

Stock solutions (1 mg/mL) of powdered nanomaterials in MilliQ water were diluted in Dulbecco’s Modified Eagle’s Medium (DMEM) (Sigma, Darmstadt). Dynamic light scattering (Malvern Zetasizer Nano series, NanoZS) was used to measure the hydrodynamic size distribution and zeta potential of the nanomaterials in culture medium prior to exposure.

### Cytotoxicity using xCELLigence

#### Maintenance of cells

Immortalised cell-lines were employed to measure toxic potencies to NMs. Cell-lines do not have all the constituents of primary cells and are genetically modified to never stop growing, nonetheless, they are a good model to assess toxic potency, especially in cases where NMs were developed as anti-cancer drugs for future use. H4IIE*-luc* rat hepatoma cells^13–15^ were obtained from University of Saskatchewan, Canada. HuTu-80 cells (HTB-40™) isolated from the human intestine were obtained from the American Type Culture Collection (Manassas, VA, USA). Both cell lines were cultured in DMEM supplemented with 10% foetal bovine serum (FBS) (Thermo Science, USA) in tissue culture dishes. Cells were maintained in a humidified incubator, with 5% CO_2_ at 37 °C. Cells were handled in a sterile laminar flow hood, which was carefully cleaned with 70% ethanol.

#### Cytotoxicity assay: exposure to nanoparticles

Cells were seeded at a density of 8.0 × 10^4^ cells/mL and left to adhere for a period of 12 h^16^. Both cell lines were exposed to 5, 25 and 50 µg/mL of Ag, Au and Au-Ag in triplicate. Unexposed cells acted as a control. Interference from NPs with the gold-plated, E-plate was monitored by adding NPs to wells containing medium, but no cells. Cytotoxicity of the two cell lines were measured independently using a real-time cell analyser; xCELLigence system RTCA single plate (SP) instrument from ACEA Biosciences with RTCA software (version 1.2.1). The software measures electrical impedance across microelectrodes on the bottom of each well in the gold-plated E-plate. The ionic state, altered by growth of cells; are measured and translated into cell index (CI) values, which are correlated in real time with growth of cells. Readings were taken every 10 min for 105 h.

### Statistical analysis

After exposure, data were normalized by use of RTCA data analysis software. Normalisation refers to the manipulation of data at a specific time point (nanomaterial treatment) which is then set as 1.0 by the software. All other values are represented as a proportion of this value. Normality was investigated by use of the Kolmogorov-Smirnov test and homogeneity of variance was assessed by use of Levene’s test (IBM, SPSS). Sample size, unequal variance and data that were not normally distributed dictated that a non-parametric test (Mann-Whitney U) had to be performed. Significance of deviations of slopes from the control slope were defined as p < 0.05^17^.

## Results and Discussion

### Characterization

Transmission electron microscopy of the NPs in MilliQ water revealed different shapes and sizes of particles. Aggregation occurred during synthesis of the Au-Ag BNP. The primary shape was spherical, however triangular and rod-like shapes were also formed. Most individual Ag-NPs and Au-NPs were more uniform and spherical with a mean diameter of 15 nm which suggested more homogenous electron densities within the volume of particles. The Au-NPs were more aggregated when compared to the more dispersed Ag-NPs. Phytochemicals present in the leaf extract were efficient at capping and stabilizing NPs.

**Figure 1:**
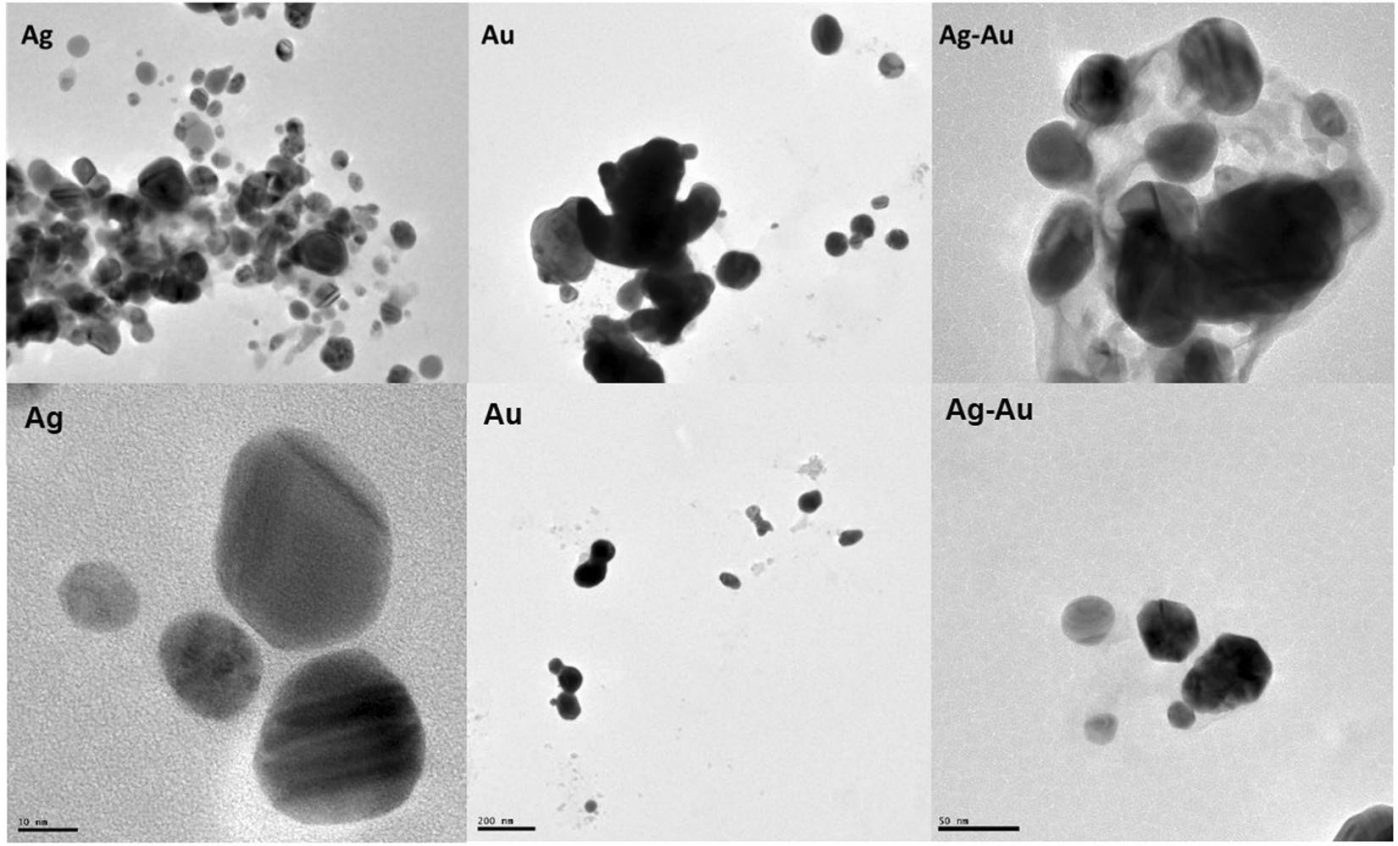
TEM images of green synthesised Ag, Au and Ag-Au bimetallic NPs.

Distribution of the synthesised NPs in the medium gave insight into their stability, solubility, motion kinetics and inherent performance in biological systems. For biological performance of nanomaterials, natural media usually contain mixed salts which would lead to increase in nanosize and sedimentation of aggregates. pH can affect dissolutions of nanomaterials by altering surface charges. Cation/anion valence concentrations of reaction media also affects stabilities of nanomaterials^18–20^. As a result, mean sizes of Au-NPs were approximately 238.2 nm, which is greater than Ag-NPs and Ag-Au BNPs, at 180.6 and 186.3 nm respectively (Table 1). Due to agglomeration experienced by the nanoparticles in DMEM medium, sizes observed during this study were greater than sizes determined by use of TEM. (Fig. 1). Both AgNPs and Ag-Au BNPs had 5.1–5.3% of the nanomaterials in the less than 10 nm range. The percentage intensity (percentage size ranges of particles distribution) is indicated in Table 1. All nanomaterials tested exhibited negative Zeta potentials. Ag-Au-NPs had a charge of −10.5 mV, while Ag had −6.84 and Au had −9.46 mV. Zeta potential greater than 30 mV or less than −30 mV are indicative of stable dispersions of nanomaterials in solution^21^. Zeta potentials observed during this study indicated that during dispersion with bath sonicator NPs formed an unstable dispersion which aggregated and eventually settled out^22^. Bioactive components of the plant extract did not affect stabilization or zeta potential.

**Table 1:** Dynamic light scattering of the nanomaterials in cell culture medium (DMEM).

NP composition	Size Z-Average (d.nm)	Size (d.nm)	% Intensity	Zeta potential (mV)
Au	238.2	147.6	66.1	−9.46
		29.72	21.6	
		8.086	12.2	
Ag	180.6	190.2	84.7	−6.84
		39.99	10.2	
		9.084	5.1	
Ag-Au bimetallic	186.3	250.3	80.1	−10.5
		40.61	13	
		8.467	5.3

### Cytotoxicity of NPs

**Figure 2:**
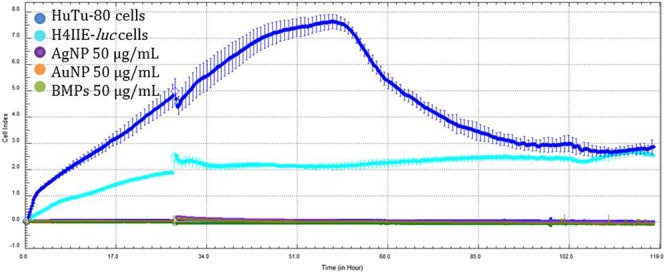
Comparison of pre normalized CI values between H4IIE-*luc* cells (cyan) and HuTu-80 cells (blue) control cells and blank wells containing only NPs indicating no particle interference.

Behaviour of NPs in biological media or determination of their toxic potency depend on material constitution or arrangement. Shapes of nanoparticles are important in determining toxicity. For instance, triangular-shaped silver nanomaterials exhibit greater toxic potency relative to spherical NPs^23^. Surface area, large ratio of surface atoms to bulk atoms results in greater reactivity and toxic potencies^24^. Potential interferences of NPs with electrical impedance were evaluated by monitoring the CI of blank wells containing only nanomaterials. These wells received the two highest concentrations (25 and 50 µg/mL) for each material to determine if interference with gold-plated wells occurred. The CI indicated no nanomaterial interferences, however HuTu-80 cells exhibited greater CI than did H4IIE*-luc* (Fig. 2). Viabilities of the two cell lines varied with differences in vulnerability ascribed to genetic differences between the two cell lines^25^. Cells were exposed to three concentrations (5, 25 or 50 µg/mL) of all the NPs prepared by use of plant extract (Au-NPs, Ag-NPs and Ag-Au-BMPs). Simplified graphs were used in figures and raw output data was included as supplementary data (Supplementary material).

**Figure 3:**
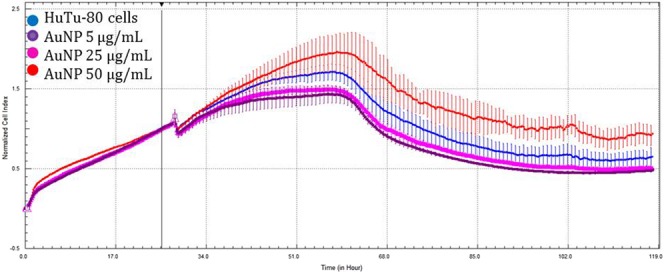
Growth curves of HuTu-80 cells exposed to three concentrations of Au-NPs for 100 h (Blue: Control; Purple: 5 µg/mL; Pink: 25 µg/mL; Red: 50 µg/mL). The line indicates addition of the nanomaterials as well as normalization time point.

**Figure 4:**
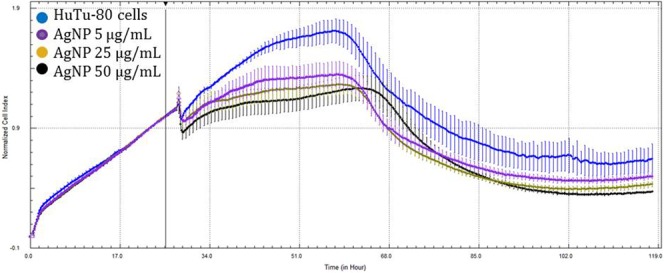
Growth curves of HuTu-80 cells exposed to three concentrations of Ag-NPs for 100 h (Blue: Control; Purple: 5 µg/mL; Yellow: 25 µg/mL; Black: 50 µg/mL). The line indicates addition of the nanomaterials as well as normalization time point.

**Figure 5:**
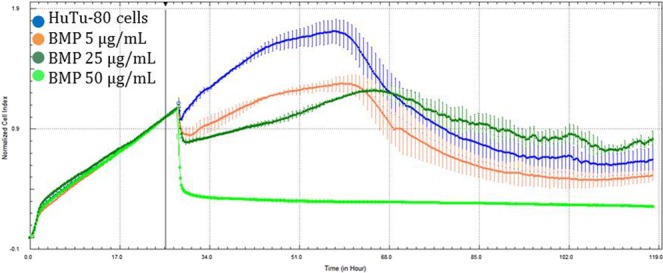
Growth curves of HuTu-80 cells exposed to three concentrations of Ag-Au-BMPs for 100 h (Blue: Control; Orange: 5 µg/mL; Dark green: 25 µg/mL; Green: 50 µg/mL). The line indicates addition of the nanomaterials as well as normalization time point.

When compared to the control, HuTu-80 cells exhibited no significant differences among the 50 µg/mL concentration of Au-NPs, although cell growth was stimulated (Fig. 3). In contrast, the greatest concentrations of Ag-NPs (Fig. 4) and Ag-Au-BMPs (Fig. 5) and second greatest concentrations of Ag-NPs (Fig. 4) and Au-NPs (Fig. 3) caused a significant decrease in cell viability (p < 0.05). Au-NPs at 5 µg/mL (Fig. 3) also caused significant cytotoxicity, but this was not the case for Ag-NPs at the same concentration (5 µg/mL) (Fig. 4) that did not significantly affect growth of cells. The two least concentrations of Ag-Au-BMPs did not significantly affect viability (Fig. 5).

**Figure 6:**
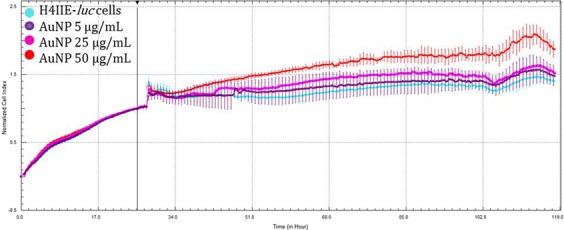
Growth of H4IIE-*luc* cells s in the presence of three concentrations of Au-NPs for 100 h (Cyan: Control; Purple: 5 µg/mL; Pink: 25 µg/mL; Red: 50 µg/mL). The line indicates addition of the nanomaterials as well as normalization time point.

**Figure 7:**
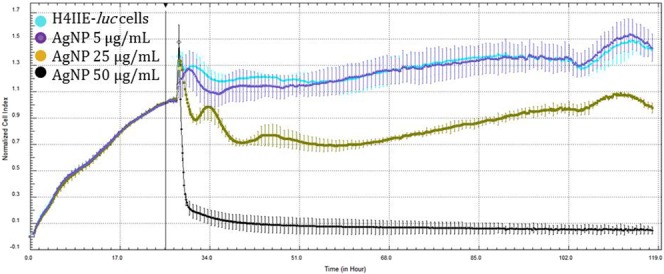
Growth curves of H4IIE-*luc* cells exposed to three concentrations of Ag-NPs for 100 h (Cyan: Control; Purple: 5 µg/mL; Yellow: 25 µg/mL; Black: 50 µg/mL). The line indicates addition of the nanomaterials as well as normalization time point.

**Figure 8:**
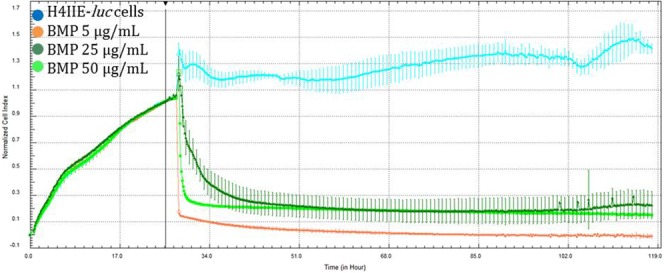
Growth curves of H4IIE-*luc* cells exposed to three concentrations of Ag-Au-BMPs for 100 h (Cyan: Control; Orange: 5 µg/mL; Dark green: 25 µg/mL; Green: 50 µg/mL). The line indicates addition of the nanomaterials as well as normalization time point.

H4IIE*-luc* cells exhibited statistically significant differences in cells exposed to the two greatest concentrations (25 and 50 µg/mL) of all three types of NPs (Figs 6–8). Au-NPs significantly stimulated growth of cells while both Ag-NPs and Ag-Au-BMPs caused significant decreases in viability of cells (Figs 6–8). The least concentration (5 µg/mL) of Ag-NPs (Fig. 7) and Au-NPs (Fig. 8) caused non-significant stimulation of growth of cells, while Ag-Au-BMPs caused a significant decrease in cell viability of H4IIE*-luc* cells.

While accumulations of Au-NPs, Ag-NPs and Au-Ag-BNPs by the HuTu-80 and H4IIE-*luc* cells were not evaluated during this study, several previous studies have investigated uptake of NPs by various types of cells^17,26^. There are many factors such as size, nature of the capping agent, zeta-potential, vehicle and coating that may influence uptake of NPs by cells^27^. Due to their small size, Ag-NPs enter mammalian cells as aggregates through endocytosis and also cross the blood-brain barrier. Upon entering cells, they are translocated to the cytoplasm and nucleus. Possible mechanisms that caused toxicity include the decrease of mitochondrial function, release of lactate dehydrogenase (LDH), cell cycle deregulation, production of reactive oxygen species (ROS) and induced apoptotic genes leading to formation of micronuclei, chromosome aberration and DNA damage^28^. AgNPs interact with the immune system and cause inflammation in treated cells^29^. Au-NPs have, however, been shown to be readily taken up into cells^16^. In contrast to the cytotoxic nature of AgNPs, AuNPs have anticancer properties. For this mechanism AuNPs target cancer cells and the tumour suppressor genes and oncogenes to induce expression of caspase-9 which is an initiator caspase involved in apoptosis^28^. Although non-immortalized cells were not included for comparison in this study, the plant nature of the compounds tested in the current study should exhibit lesser toxic potency compared to non-cancerous cells as well as those tested. This is due to the antioxidant properties of plant-based molecules that results in greater toxicity to cancerous cells and lesser toxicity to healthy cells by expression of apoptotic molecules^30^. Targeted treatment using NMs for anticancer therapy has proven to show promise however once NMs are released and can come in contact with normal cells they will be further altered by interactions with biomolecules, changes in zeta potential and dissolution rates. All changes that NMs undergo can therefore affect their toxicity to cells. Adaption of BNPs, by altering the surface alloy, can increase their ability for cancer therapy by decreasing toxic effects on normal cells^28^.

Since surface charges of the various NPs were all negative, uptake can be related to size, which was within a similar range, and the surface-core makeup of various NPs. Since aggregations of Au-Ag-BNPs were observed during this study, particles could have been taken up as clusters, a phenomenon that has also been observed previously^31^ NPs can be accumulated by cells by various mechanisms depending on sizes of aggregations. As reported previously aggregated NPs can be accumulated by a combination of macro-pinocytosis and caveolae-mediated endocytosis^32^. Larger particles result larger loads, which in turn, as observed for Au-Ag-BNPs results in greater toxic potency. States of NPs in media might also affect accumulation into and clearance from cells^25^. Au-Ag-BNPs exhibited greater toxic potency to both cell lines. Accumulation of NPs into cells could be aided by the Au surface coating by a Trojan horse effect. Once Au-Ag-BNPs entered cells, it is broken down and the consequent release of Ag occurs, which can result in greater toxicity as seen in monometallic Ag exposure.

In solutions containing Ag-NPs, zero-valent silver (Ag°) sometimes occurs with forms of ionic Ag, either from partial reduction of precursors or oxidation of silver NPs to release Ag^+^. Such situations were suggested data from the powder X-ray diffraction^33^. Cationic silver (Ag^+^) has been reported to have potentially greater toxic potency compared to Ag^34^. Toxicological effects vary as a function of oxidation state and also dissolution characteristics of NPs^35^. Silver sulphide (AgS) is less bioavailable and less toxic to living organisms^36^. Toxicological nature of Ag NPs in this research could be due to the coexistence of Ag_2_O in both Ag-NPs and Ag-Au NPs. It has also been reported that toxicological profiles or biological behaviours of NP can vary, depending on the substrate used^37^.

## Conclusions

Unique properties of NPs instil them with beneficial properties. The use of plant extracts for the synthesis of nanomaterials can present interesting and useful properties. These “green” extracts that serve as substrates in syntheses of NPs can significantly affect properties and behaviours of NPs. Nanoparticles obtained from plant extracts might be less expensive and more ecologically friendly, than the conventional, less natural, ones. Due to increasing production and widespread usage of nanomaterials especially in biological applications, assessments of potential effects of nanoparticles in cells were necessary. Zeta potentials revealed unstable natures of nanoparticles, which might have resulted from aggregation of particles. Not all biologically synthesized nanomaterials are necessarily safe. AuNPs synthesized from golden rod extract exhibited lesser toxic potency than NPs synthesized without plant leaf extracts. Thus NPs synthesized in the presence of plant extract might be useful as theranostic agents. Mechanisms of reactions of nanomaterials in media are complex and more investigation is needed to establish baseline information before widespread applications. Uses of normal (non-cancerous) cell cultures are also recommended in further testing of the toxicity of the bimetallic nanoparticles prepared in this study, as they are closer to an *in vivo* situation and should be included in tests to validate these compounds for clinical use.

## Supplementary information

Supplementary information
