# Supplementary material for: Cytotoxicity of Ag, Au and Ag-Au bimetallic nanoparticles prepared using golden rod (Solidago canadensis) plant extract
Source: Sci Rep. 2019 Mar 12;9:4169. doi: 10.1038/s41598-019-40816-y (PMC6414615; doi:10.1038/s41598-019-40816-y)
Supplement: Supplementary file 1 — Supplementary information [file 41598_2019_40816_MOESM1_ESM.pdf]

Cytotoxicity of Ag, Au and Ag-Au bimetallic nanoparticles prepared using golden rod (*Solidago canadensis*) plant extract

[1]Tarryn L. Botha*, [2,3,4] Elias E. Elemike, [1]Suranie Horn, [2,3]Damian C. Onwudiwe, [5,6,7,8] John P. Giesy, and [1] Victor Wepener

*Corresponding author: Tarryn L Botha

*E-mail: 26964546@nwu.ac.za

1. Water Research Group, Unit for Environmental Sciences and Management, Potchefstroom Campus, North-West University, Private Bag X6001, Potchefstroom, 2520, South Africa
2. Material Science Innovation and Modelling (MaSIM) Research Focus Area, Faculty of Agriculture, Science and Technology, North-West University, Mafikeng Campus, Private Bag X2046, Mmabatho 2735, South Africa.
3. Department of Chemistry, School of Mathematics and Physical Sciences, Faculty of Agriculture, Science and Technology, North-West University, Mafikeng Campus, Private Bag X2046, Mmabatho 2735, South Africa.
4. Department of Chemistry, College of Science, Federal University of Petroleum Resources, P.M.B 1221, Effurun, Delta State, Nigeria.
5. Department of Veterinary Biomedical Sciences and Toxicology Centre, University of Saskatchewan, Saskatchewan, Canada
6. Department of Zoology, and Center for Integrative Toxicology, Michigan State University, East Lansing, MI, USA
7. School of Biological Sciences, University of Hong Kong, Hong Kong, SAR, China
8. State Key Laboratory of Pollution Control and Resource Reuse, School of the Environment, Nanjing University, Nanjing, People’s Republic of China

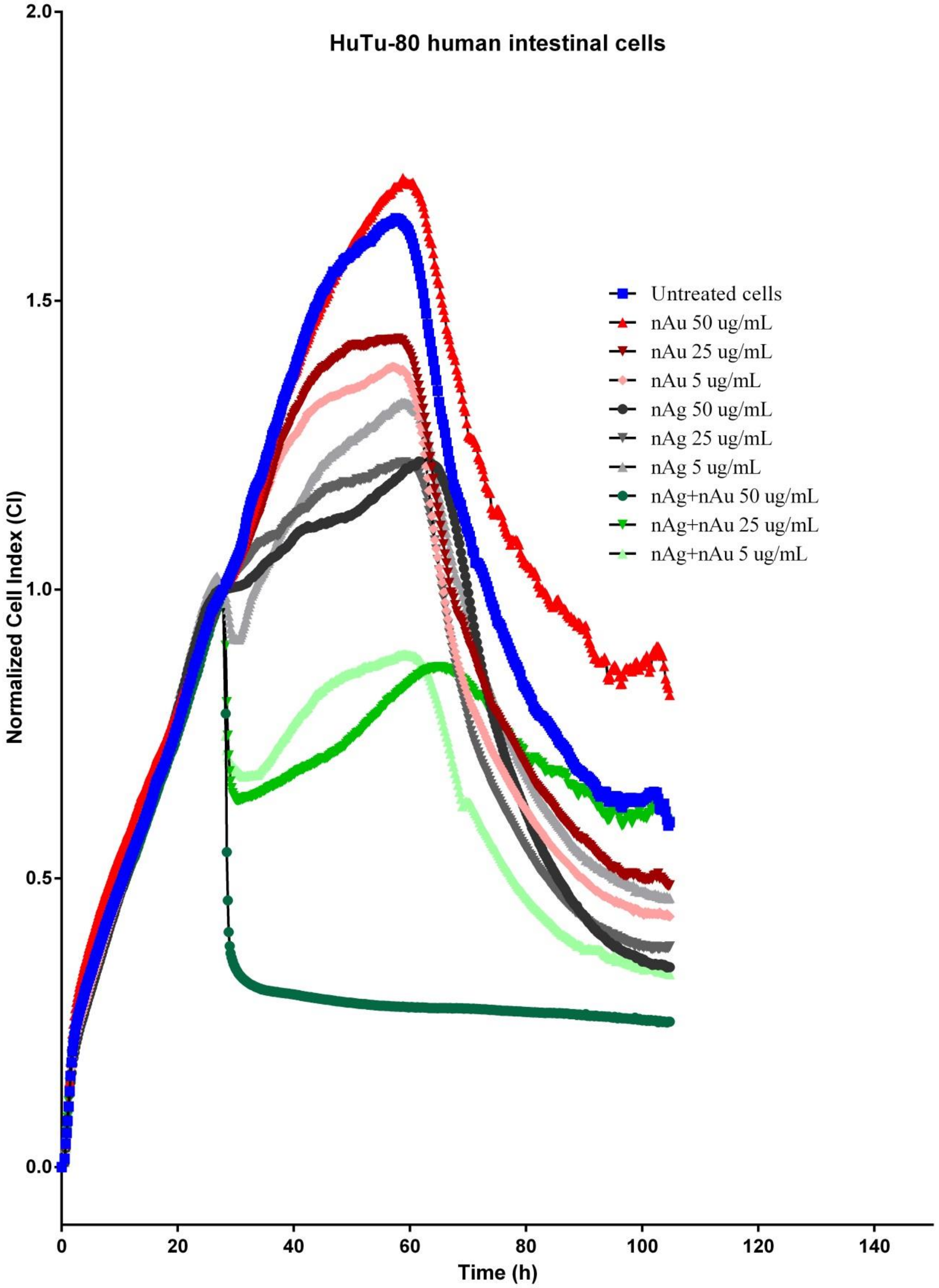

Supplementary Fig 1. Simplified mean growth of HuTu cells in the presence of three concentrations of Au-NPs, Ag-NPs and Ag-Au-BMPs for 100 h.

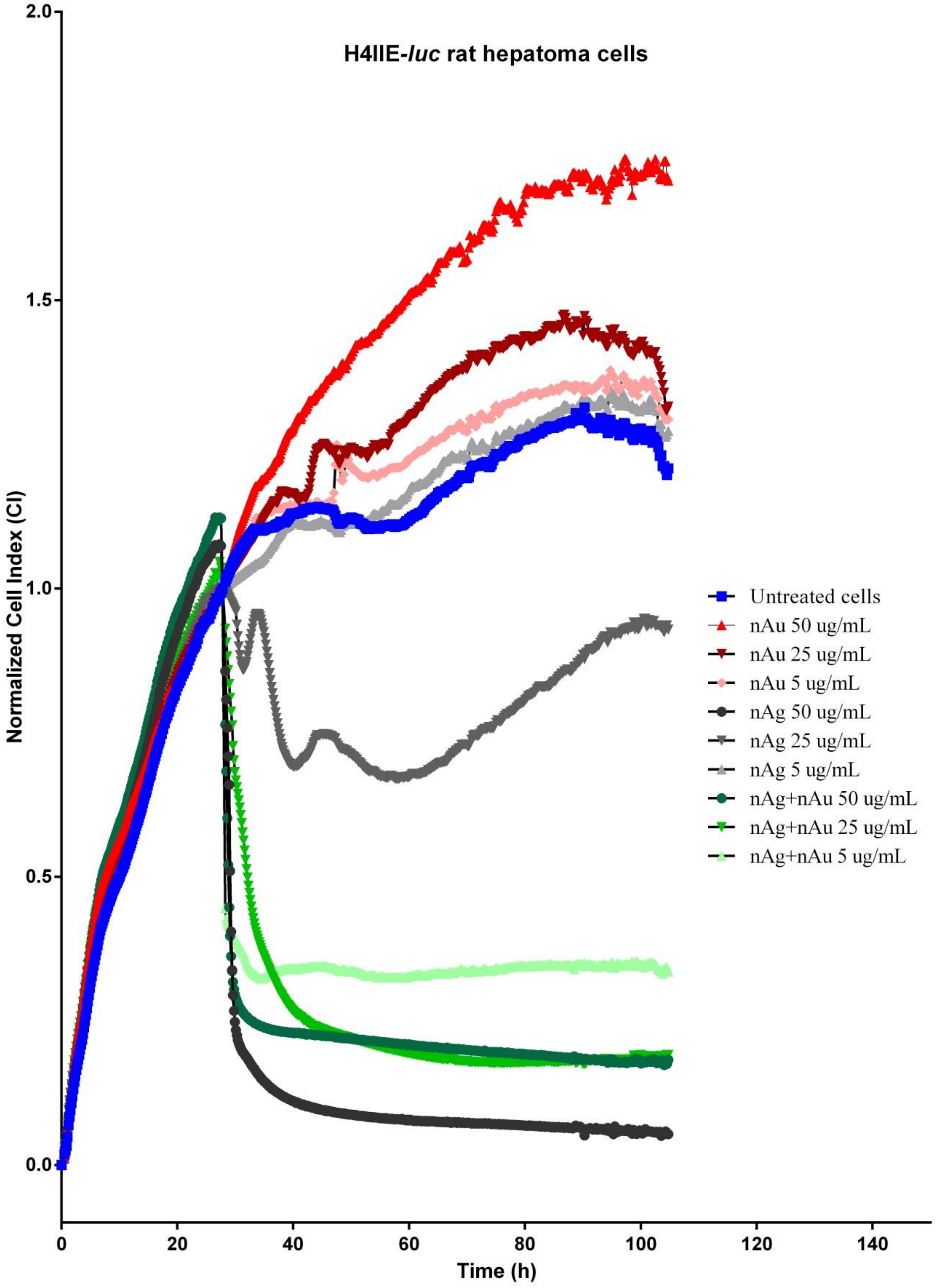

Supplementary Fig 2. Simplified mean growth curves of H4IIE-*luc* cells exposed to three concentrations of Au-NPs, Ag-NPs or Ag-Au-BMPs for 100 h.
